# Physical activity and subclinical atherosclerosis in chronic Chagas disease: a cross-sectional study

**DOI:** 10.3389/fmed.2026.1793058

**Published:** 2026-05-15

**Authors:** Erica Maria Diferenz, Roberto Magalhães Saraiva, Andréa Rodrigues da Costa, Whesley Tanor Silva, Henrique Silveira Costa, Mauro Augusto dos Santos, Alejandro Marcel Hasslocher-Moreno, Fernanda de Souza Nogueira Sardinha Mendes, Tatiana Rehder Gonçalves, Luiz Fernando Rodrigues, Daniel Arthur Barata Kasal, Mauro Felippe Felix Mediano

**Affiliations:** 1 Department of Research and Education, National Institute of Cardiology, Rio de Janeiro, Brazil; 2 Evandro Chagas National Institute of Infectious Diseases, Oswaldo Cruz Foundation, Rio de Janeiro, Brazil; 3 Department of Physiotherapy, Healthy and Biological Sciences Faculty, Federal University of Vales do Jequitinhonha and Mucuri, Diamantina, Brazil; 4 Laboratory of Human Performance, Rio de Janeiro, Brazil; 5 Hésio Cordeiro Institute of Social Medicine, State University of Rio de Janeiro, Rio de Janeiro, Brazil; 6 Department of Internal Medicine, State University of Rio de Janeiro, Rio de Janeiro, Brazil

**Keywords:** cardiovascular health, Chagas disease, lifestyle, physical activity, subclinical atherosclerosis

## Abstract

**Introduction:**

The clinical–epidemiological profile of Chagas disease (CD) has changed over the past decades, leading to decreased levels of physical activity (PA), which may be associated with changes in markers of subclinical atherosclerosis. This study aimed to investigate the association between PA levels and carotid intima–media thickness (CIMT), the presence of carotid atherosclerotic plaque (CAP), and the amount of epicardial adipose tissue (EAT) in patients with chronic CD.

**Methods:**

This cross-sectional study included patients with chronic CD. The PA level was determined using the short version of the International Physical Activity Questionnaire (IPAQ-short). CIMT and CAP were assessed using Doppler ultrasound of the carotid arteries. The amount of EAT was assessed using transthoracic echocardiography. Linear and binomial logistic regression models were used.

**Results:**

The median age of the 349 participants was 62 years (54.0–69.0), 56.5% were women, and 79.5% were non-white, with 67.6% having <9 years of schooling. The most common clinical form of CD was the cardiac form without heart failure (HF) (53.9%). Median CIMT values were 0.65 mm (left) and 0.66 mm (right). CAP was present in 46.4% of participants, and the median EAT was 5.0 mm. No statistically significant association was observed between PA levels and CIMT, the presence of CAP, or the amount of EAT.

**Conclusion:**

PA levels were not associated with the markers of subclinical atherosclerosis in individuals with chronic CD.

## Introduction

Chagas disease (CD) is a parasitic infectious disease caused by the flagellated protozoan *Trypanosoma cruzi,* affecting approximately 8 million people worldwide (1, 2). The main clinical manifestation of chronic CD is chronic Chagas cardiomyopathy (CCC), which occurs in 20–40% of infected individuals and is associated with high levels of morbidity and mortality (3, 4). This condition is characterized by clinical manifestations such as heart failure, thromboembolism, stroke, arrhythmias, cardiac conduction system block, and sudden death (4). The main non-cardiac complications of CD are megaesophagus and megacolon, which can occur in approximately 10–20% of cases (5).

Over the past decades, improvements in treatment have allowed many individuals with CD to live longer, resulting in an aging patient population (5). In parallel, many individuals with CD have migrated from rural areas to major urban centers, leading to lifestyle changes (6), such as reduced levels of physical activity (PA) (7), which, in turn, increase the risk of atherosclerosis and, consequently, cardiovascular diseases (8). Despite the widely known benefits of PA, low levels of PA are found in individuals with chronic health conditions, including CD (9). Considering that low PA levels are an important risk factor for cardiovascular health, it is necessary to evaluate whether PA levels are associated with markers of subclinical atherosclerosis, such as common carotid artery intima–media thickness (CIMT), carotid atherosclerotic plaques (CAPs), and the amount of epicardial adipose tissue (EAT), in patients with chronic CD. To the best of our knowledge, no previous study has explored this relationship in this population. Thus, this study aimed to investigate the association between PA levels and markers of subclinical atherosclerosis in individuals with chronic CD. We hypothesized that, among individuals with chronic CD, lower levels of PA would be associated with worse markers of subclinical atherosclerosis.

## Methods

### Study design and population

This is an observational cross-sectional study conducted from June 2014 to March 2017, including participants of both sexes aged >18 years, with chronic CD diagnosed by two concurrently positive serological tests (enzyme-linked immunosorbent assay and indirect immunofluorescence). All patients were under clinical follow-up at the outpatient center of the Evandro Chagas National Institute of Infectious Disease/Oswaldo Cruz Foundation, a national reference center for the treatment and research of infectious and tropical diseases in Rio de Janeiro, Brazil. Participants were excluded if they presented with heart disease of non-Chagas etiology, autoimmune disorders, cancer, other infectious diseases at the time of study recruitment, severe cognitive impairments, current use of chronic anti-inflammatory agents or corticosteroids, or pregnancy.

### Ethical consideration

The study was conducted in accordance with the Declaration of Helsinki, revised in 2013, and with the Brazilian National Health Council Resolution 466/2012. The study was approved by the Institutional Review Board of the Evandro Chagas National Institute of Infectious Disease (CAAE: 22985313.8.0000.5262). All participants received information about the objectives and procedures of the study and agreed to participate by signing an informed consent form.

### Study procedures and measurement tools

Patients were invited to participate during their regular clinic visits and were required to complete the study procedures in two visits within 1 month. During the first visit, patients signed the informed consent form and underwent assessments of sociodemographic, clinical, and lifestyle-related variables. During the second visit, Doppler ultrasound of the carotid arteries and transthoracic echocardiography were performed to assess CIMT, the presence of CAP, and the amount of EAT. Trained staff administered the questionnaires and performed the anthropometric measurements. The same cardiologist, blinded to the PA data, performed all Doppler ultrasound examinations of the carotid arteries and the transthoracic echocardiogram using a GE Medical Systems Vivid 7 vascular ultrasound device with a 7–10-MHz frequency transducer, as guided by an experienced echocardiographer who was also blinded to the PA data.

### Physical activity assessment

The level of PA was determined using the short version of the International Physical Activity Questionnaire (IPAQ), adapted and validated for use in the Brazilian population (10, 11). The total volume of PA was calculated using metabolic equivalent of the task (MET)-min/week and stratified into tertiles for analytical purposes (high, intermediate, and low). To calculate the level of PA in MET-min/week, the MET value of each activity was multiplied by the total duration of the activity in minutes and then by the number of times the activity was performed per week. The MET values assigned were 3.3 METs for walking, 4.0 METs for moderate activities, and 8.0 METs for vigorous activities. The total volume of PA was calculated by summing the MET-min/week from walking, moderate activities, and vigorous activities, while the volume of moderate-to-vigorous PA was calculated by summing only the moderate and vigorous activities. The data were limited to a maximum time of 180 min per day, allowing a maximum of 21 h of PA per week for each category, as recommended in the IPAQ scoring protocol guidelines (12). The level of PA was also assessed as a continuous variable, considering the variation of each 100 MET-min/week, which represents approximately 20 min of brisk walking per week, or approximately 3 min of brisk walking per day, every day of the week.

### Carotid intima–media thickness

The CIMT was assessed by echo-Doppler ultrasound of the distal common carotid artery, 2 cm from the carotid bifurcation, performed in both arteries (right and left) using a 7–10-MHz frequency transducer in a two-dimensional (2D) mode, with longitudinal and transverse sections. The CIMT was obtained by measuring the distance between two echogenic lines representing the lumen–intima and media–adventitia interfaces of the arterial wall (13).

### Presence of carotid atherosclerotic plaque

The presence of CAP was assessed by Doppler ultrasound of the common and internal carotid arteries on both the right and left sides. Examinations were performed in 2D mode using transverse and longitudinal sections with a 7–10 MHz frequency transducer. The presence of CAP was defined as a focal wall thickening exceeding 50% of the surrounding carotid segment or a CIMT measurement greater than 1.5 mm (13).

### Epicardial adipose tissue

The amount of EAT was assessed using a 3.5-MHz frequency transducer in two-dimensional mode. EAT thickness was calculated by measuring the echo-free space between the myocardium of the free wall of the right ventricle and the visceral pericardium at end-systole, perpendicular to the aortic annulus, in the parasternal echocardiographic window of the long axis (14).

### Covariates

Sociodemographic, lifestyle, and clinical covariates were obtained to characterize the study population and address potential confounding factors in the studied associations. Information on age, sex, schooling, and ethnicity was obtained during the interviews. Age was calculated as the difference between the date of the interview and the date of birth and was considered as a continuous variable. Based on the formal years of study, schooling was categorized into < 9 years, 9–12 years, and > 12 years. Ethnicity was self-reported and categorized as white and non-white (Black, mixed ethnicity, Asian, and Indigenous individuals). Smoking status was classified as current/former smoker or never smoker. Alcohol consumption was categorized as current/former drinker or never drinker. Sleep hours were determined through a direct question and treated as a continuous variable. Food consumption was assessed using a 24-h recall that consisted of the identification and quantification of all food and beverages consumed on the day before the interview. Macronutrients were calculated using Diet Win Professional Version 2008 software.

Comorbidities (hypertension, diabetes, dyslipidemia, and obesity) were obtained using information from medical records and anthropometric measurements during the clinical evaluation. The clinical classification of CD was based on clinical, electrocardiographic, and digestive examinations, and participants were categorized as having the indeterminate form, CCC without heart failure, CCC with heart failure, or the digestive form, in accordance with the Brazilian Consensus on CD (15).

### Data analysis

For descriptive data analysis, medians were used, with an interquartile range (25th–75th) for continuous variables and absolute frequencies and percentages for categorical variables. The comparison of clinical, sociodemographic, and lifestyle characteristics between tertiles of PA levels was performed using Cuzick’s trend tests for continuous variables and Jonckheere–Terpstra test for categorical variables. Linear regression and binomial logistic regression models were used to assess the association between PA levels (considered as a categorical variable [PA tertiles] and as a continuous variable [increments of 100 MET-min/week]) and CIMT and EAT (as continuous variables), as well as the presence of CAP (a binary categorical variable). Both unadjusted and adjusted models were fitted to account for potential confounding variables. The minimum set of adjustment variables was identified using a directed acyclic graph (DAG) and included age, sex, ethnicity, schooling, smoking, hypertension, diabetes mellitus, dyslipidemia, food intake (carbohydrates, lipids, and proteins), and stages of CCC (without CCC, CCC without HF, or CCC with HF), as illustrated in Supplementary Figure 1, created using the Dagitty program (available at https://dagitty.net/).

## Results

Of the 397 patients initially included, 48 were excluded for the following reasons: 6 had other infectious diseases, 3 had autoimmune diseases, 6 had cancer, 8 had non-Chagas cardiomyopathy, 5 were using anti-inflammatory drugs or corticosteroids, 8 were lost to follow-up, and 12 had missing data for CIMT, CAP, or EAT, resulting in a final analytic sample of 349 participants (Supplementary Figure 2).

Table 1 shows the characteristics of patients, both overall and stratified by tertiles of total PA. The median age was 62 years, with approximately half (56.5%) of them being women and a predominance of <9 years of schooling (67.6%). The prevalence of comorbidities was 67.6% for hypertension, 53.9% for dyslipidemia, and 25.5% for obesity. The most common clinical form of CD was the cardiac form without HF, at 53.9%. The median total PA level was 1,548.0 MET-min/week, while moderate-to-vigorous PA had a median of 720.0 MET-min/week. The median CIMT was 0.65 mm on the left side and 0.66 mm on the right side. CAP was present in 46.4% of participants. The median EAT was 5.0 mm. Considering the characteristics according to the tertiles of PA, there was a trend toward lower age with higher levels of PA, along with reduced CIMT values and a lower proportion of patients with CAP (Table 1).

**Table 1:** Characteristics of patients stratified by tertiles of total activity level (*n* = 349).

Variables	Total (*n* = 349; 100%)	Total PA tertiles^†^			*p*-value*
		Lowest (*n* = 117; 33.6%)	Intermediate (*n* = 116; 33.2%)	Highest (*n* = 116; 33.2%)	
Age in years (median; IQR)	62.0 (54.0–69.0)	64.0 (56.0–71.0)	63.0 (53.0–68.5)	60.0 (51.5–65.0)	<0.001
Women (%; *n*)	56.5 (197)	59.0 (69)	54.3 (63)	56.0 (65)	0.65
White race (%; *n*)	21.5 (75)	21.4 (25)	22.4 (26)	20.7 (24)	0.90
Schooling (%; *n*)					
<9 years	67.6 (236)	70.9 (83)	72.4 (84)	59.5 (69)	0.06
9–12 years	18.6 (65)	16.2 (19)	17.2 (20)	22.4 (26)	0.22
>12 years	13.8 (48)	12.8 (15)	10.3 (12)	18.1 (21)	0.24
Sleep hours (median; IQR)	7.0 (6.0–8.0)	7.0 (5.0–8.0)	6.0 (5.3–8.0)	7.0 (6.0–8.0)	0.74
Weight in kg (median; IQR)	67.5 (59.4–78.6)	66.3 (58.5–76.3)	67.9 (60.2–79.4)	68.3 (61.2–78.3)	0.22
Height in m (median; IQR)	1.60 (1.53–1.65)	1.57 (1.53–1.63)	1.60 (1.53–1.65)	1.60 (1.55–1.66)	0.99
BMI Kg/m^2^ (median; IQR)	26.8 (23.9–30.0)	26.3 (23.1–30.9)	27.0 (24.3–30.2)	27.0 (24.0–29.8)	0.70
Waist circumference in cm (median; IQR)	89.9 (82.1–98.3)	90.5 (81.2–97.2)	90.5 (81.2–97.2)	89.7 (80.8–98.7)	0.80
Number of comorbidities (median; IQR)	2.0 (1.0–2.0)	2.0 (1.0–3.0)	2.0 (1.0–2.0)	2.0 (1.0–2.0)	0.15
Hypertension (%; *n*)	67.6 (236)	66.7 (78)	71.6 (83)	64.7 (75)	0.74
Diabetes mellitus (%; *n*)	20.9 (73)	23.9 (28)	19.8 (23)	19.0 (22)	0.35
Dyslipidemia (%; *n*)	53.9 (188)	58.1 (68)	55.2 (64)	48.3 (56)	0.13
Obesity (%; *n*)	25.5 (89)	27.4 (32)	26.7 (31)	22.4 (26)	0.38
Previous use of benznidazole (%; *n*)	9.2 (32)	7.7 (9)	10.3 (12)	9.5 (11)	0.63
Smoking (%; *n*)	46.7 (163)	49.6 (58)	51.7 (60)	38.8 (45)	0.10
Alcohol consumption (%; *n*)	39.5 (138)	44.4 (52)	37.0 (43)	37.0 (43)	0.24
Food consumption in grams (median; IQR)					
Carbohydrates	175.9 (137.6–240.2)	175.0 (125.7–238.8)	176.0 (138.8–237.0)	184.6 (140.4–246.3)	0.43
Lipids	34.9 (25.1–48.6)	35.0 (27.3–49.0)	32.7 (24.9–43.1)	40.0 (21.7–51.6)	0.76
Proteins	61.9 (45.8–81.2)	48.3 (61.1–79.5)	61.7 (44.0–81.4)	65.5 (46.8–87.5)	0.43
Fiber	16.6 (11.3–23.4)	15.9 (11.2–23.0)	16.7 (10.9–24.4)	17.0 (11.7–23.0)	0.58
Clinical form (%; *n*)					
Indeterminate	26.9 (94)	22.2 (26)	28.4 (33)	30.2 (35)	0.17
Cardiac	57.0 (199)	53.9 (63)	60.3 (70)	56.9 (66)	0.63
Digestive	3.7 (13)	4.3 (5)	4.3 (5)	2.6 (3)	0.49
Mixed	12.3 (43)	19.7 (23)	6.9 (8)	10.3 (12)	0.03
CCC stage (%; *n*)					
Without CCC	30.7 (107)	26.5 (31)	32.8 (38)	32.8 (38)	0.29
CCC without HF (stages A, B1, and B2)	53.9 (188)	57.2 (67)	49.1 (57)	55.2 (64)	0.74
CCC with HF (stages C and D)	15.5(54)	16.2 (19)	18.1 (21)	12.0 (14)	0.38
Carotid intima–media thickness in mm					
Left	0.65 (0.58–0.76)	0.68 (0.59–0.80)	0.65 (0.57–0.76)	0.61 (0.58–0.71)	0.05
Right	0.66 (0.59–0.77)	0.68 (0.60–0.80)	0.66 (0.60–0.78)	0.61 (0.57–0.73)	0.01
Carotid atherosclerotic plaque (%)	46.4 (162)	52.1 (61)	47.4 (55)	39.7 (46)	0.05
Epicardial adipose tissue in mm	5.0 (4.0–6.0)	5.0 (4.0–6.0)	5.0 (4.0–6.0)	4.0 (3.0–6.0)	0.33

PA: physical activity; MET: metabolic equivalent of the task; IQR: interquartile range of 25–75%; BMI: body mass index; CCC: chronic Chagas disease; HF: heart failure. * Cuzixk’s test for trends in continuous variables/Jonckheere–Terpstra’s test for trends in categorical variables. ^†^ PA total MET-min/week (median; IQR: 25–75%): total: 1548.0 (690.0–3066.0); low: 462.0 (120.0–690.0); intermediate: 1561.5 (1271.0–1803.5); high: 4290.0 (3132.5–5733.0).

Table 2 shows the characteristics of CIMT, CAP, and EAT stratified by sex, age, and CCC stage groups. Overall, older individuals (≥65 years) showed higher CIMT values both on the left (0.61 vs. 0.71 mm; *p* < 0.0001) and the right (0.61 vs. 0.73 mm; *p* < 0.0001) sides and greater EAT (4.0 vs. 6.0 mm; p < 0.0001) than those aged < 65 years. Additionally, reduced EAT values were observed among individuals with CCC with heart failure compared to those without CCC and those with CCC without heart failure (4.0 vs 5.0 vs 5.0 mm, respectively; *p* < 0.04) (Table 2).

**Table 2:** Characteristics of CIMT, CAP, and EAT stratified by groups of sex, age, and stages of CCC (*n* = 349).

Variables	Sex		*p*-value	Age		*p*-value	CCC stage			*p*-value
	Men	Women		<65 years	≥65 years		Without CCC	CCC without HF	CCC with HF	
CIMT (mm)										
Left	0.68 (0.59–0.80)	0.63 (0.58–0.74)	0.10	0.61 (0.56–0.70)	0.71 (0.60–0.90)	<0.001	0.61 (0.56–0.74)	0.67 (0.60–0.79)	0.66 (0.58–0.76)	0.19
Right	0.63 (0.59–0.78)	0.66 (0.58–0.76)	0.70	0.61 (0.56–0.70)	0.73 (0.64–0.88)	<0.001	0.63 (0.58–0.73)	0.67 (0.60–0.79)	0.64 (0.58–0.73)	0.46
CAP (%)	44.1 (67)	48.2 (95)	0.44	29.6 (63)	72.8 (99)	<0.001	40.2 (43)	52.7 (99)	37.0 (20)	0.66
EAT (mm)	4.5 (3.0–6.0)	5.0 (4.0–6.0)	0.07	4.0 (3.0–6.0)	6.0 (4.0–7.0)	<0.001	5.0 (3.0–6.0)	5.0 (4.0–6.0)	4.0 (3.0–5.0)	0.04

CIMT: carotid intima–media thickness; CAP: carotid atherosclerotic plaque; EAT: epicardial adipose tissue in mm.

**Figure 1:**
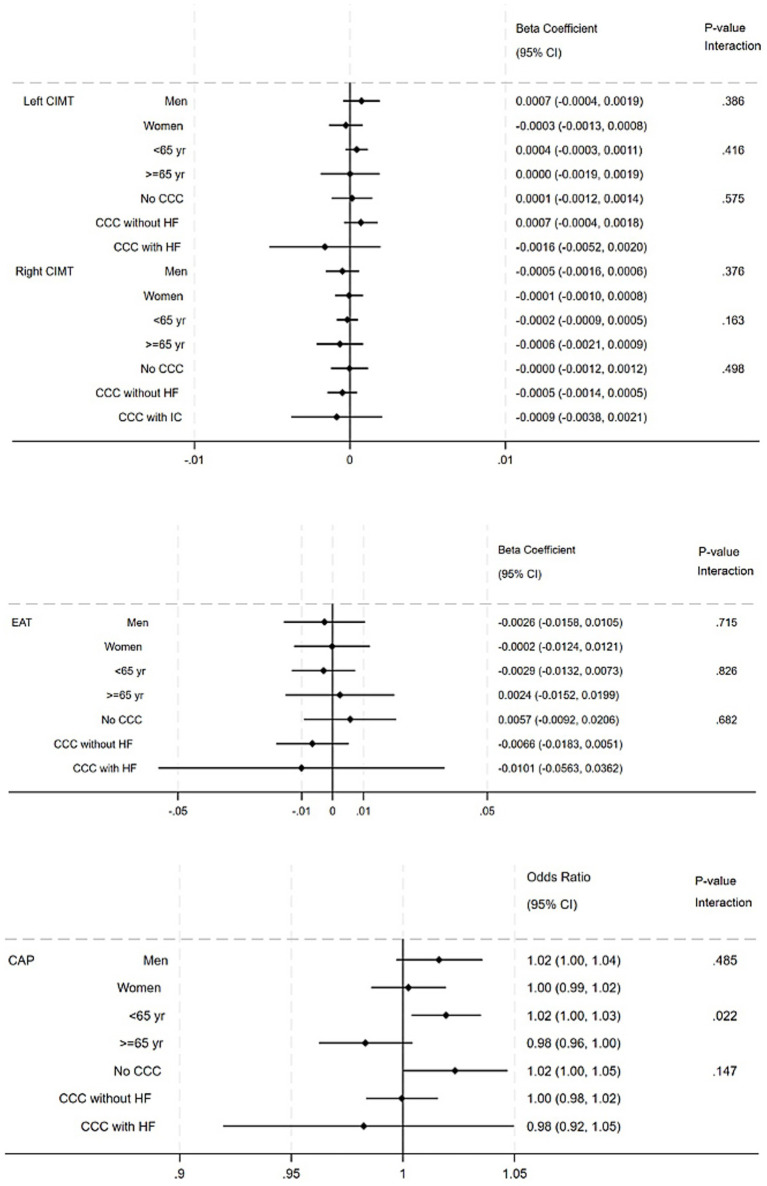
Forest plots for the adjusted associations between total PA levels and the amount of EAT, CIMT (left and right), and the presence of CAP in the different subgroups of sex, age, and CCC stage.

Table 3 shows the estimates for the association between total PA levels and CIMT, the presence of CAP, and the amount of EAT. In the unadjusted analysis, a statistically significant association was observed between the highest PA tertile and lower left (*β* = −0.49; 95%CI -0.93 to −0.005) and right (β = −0.05; 95%CI -0.09 to −0.01) CIMT. However, these associations did not remain statistically significant after adjusting for potential confounding variables (Table 3). Similar results were observed for the adjusted associations between total PA levels and CIMT, the presence of CAP, and the amount of EAT in the different subgroups of sex, age, and CCC stage (Figure 1).

**Table 3:** Estimates for the association of total PA level with CIMT, the amount of EAT, and the presence of CAP in patients with CD (*n* = 349).

	Total PA tertiles (MET-min/week)			
	Low (*n* = 117)	Intermediate (*n* = 116)	High (*n* = 116)	Continuous Per 100 MET-min/week (*n* = 349)
	β (95% CI)			
**CIMT left**				
Not adjusted	Reference	−0.29 (−0.73 to +0.15)	−0.49 (−0.93 to −0.005)	−0.0004 (−0.0001 to +0.0004)
Adjusted*		−0.01 (−0.05 to +0.03)	−0.02 (−0.06 to +0.03)	+0.0002 (−0.001 to +0.001)
**CIMT right**				
Not adjusted	Reference	−0.01 (−0.05 to +0.03)	−0.05 (−0.09 to −0.01)	−0.001 (−0.002 to +0.0003)
Adjusted*		+0.01 (−0.03 to +0.05)	−0.01 (−0.05 to +0.02)	−0.0003 (−0.001 to +0.0004)
**EAT**				
Not adjusted	Reference	+0.09 (−0.41 to +0.59)	−0.15 (−0.65 to + 0.35)	−0.01 (−0.02 to +0.001)
Adjusted*		+0.21 (−0.24 to +0.68)	+0.16 (−0.30 to +0.63)	−0.002 (−0.01 to +0.01)
	OR (95%CI)			
**CAP**				
Not adjusted	Reference	0.82 (0.49 to 1.39)	0.60 (0.36 to 1.01)	0.99 (0.99 to 1.01)
Adjusted*		1.19 (0.63 to 2.21)	1.04 (0.55 to 1.96)	1.00 (0.99 to 1.02)

	Moderate-to-vigorous PA Tertiles (MET-min/week)			
	Low (*n* = 117)	Intermediate (*n* = 116)	High (*n* = 116)	Continuous Per 100 MET-min/week (*n* = 349)
	β (95% CI)			
**CIMT left**				
Not adjusted	Reference	−0.008 (−0.05 to +0.04)	−0.04 (−0.09 to +0.001)	−0.0006 (−0.002 to +0.0004)
Adjusted*		+0.01 (−0.03 to +0.06)	−0.01 (−0.05 to +0.03)	+0.0002 (−0.0008 to +0.001)
**CIMT right**				
Not adjusted	Reference	−0.01 (−0.05 to +0.03)	−0.05 (−0.09 to −0.06)	−0.001 (−0.002 to −0.0002)
Adjusted*		+0.02 (−0.02 to +0.05)	−0.006 (−0.04 to +0.03)	−0.0001 (−0.001 to +0.001)
**EAT**				
Not adjusted	Reference	+0.07 (−0.42 to +0.60)	−0.25 (−0.75 to +0.26)	−0.01 (−0.02 to +0.0005)
Adjusted*		+0.30 (−0.16 to +0.76)	+0.04 (−0.43 to +0.51)	−0.005 (−0.02 to +0.01)
	OR (95%CI)			
**CAP**				
Not adjusted	Reference	0.63 (0.38 to 1.05)	0.71 (0.42 to 1.20)	0.99 (0.98 to 1.01)
Adjusted*		0.88 (0.47 to 1.63)	1.26 (0.67 to 2.38)	1.01 (0.99 to 1.03)

*Adjusted for age, sex, race, schooling, smoking, hypertension, diabetes, dyslipidemia, food intake (carbohydrates, lipids, and proteins), and stages of CCC (without CCC, CCC without HF, and CCC with HF); CIMT: carotid intima–media thickness; CAP: carotid atherosclerotic plaque; EAT: epicardial adipose tissue.

Table 3 shows the estimates for the association of moderate-to-vigorous PA levels with CIMT, the presence of CAP, and the amount of EAT. In the unadjusted analyses, a statistically significant association was observed between the highest tertile of PA and lower CIMT in the right carotid artery (*β* = −0.05; 95% CI -0.09 to −0.06), as well as an association for each increment of 100 MET × minutes per week (β = −0.001; 95% CI -0.002 to −0.0002). However, these associations were not statistically significant after adjustments for potential confounding variables (Table 3).

## Discussion

To the best of our knowledge, this is the first study to investigate markers of subclinical atherosclerosis (CIMT, CAP, and EAT) in patients with CD and to evaluate their relationship with PA levels. Our findings revealed CIMT values similar to those reported in other populations in the literature, particularly among asymptomatic adults from low- and middle-income countries (16). These results are also consistent with data from the Brazilian population, as demonstrated by the ELSA-Brasil study (17). However, despite the inflammatory characteristics of CD, the values observed in the present study are lower than those found in other chronic inflammatory diseases, such as rheumatoid arthritis (18). This difference may be attributed to the distinct inflammatory profiles between these conditions, as, in chronic CD, inflammation tends to be less systemic compared with that of rheumatoid arthritis (19, 20).

Regarding the prevalence of CAP, we observed a lower prevalence in our sample compared with studies from both Brazil and other countries, despite a similar age range (21–23). In this context, we could speculate that CD has unique features that differentially influence the development of atherosclerosis compared to other populations. Some studies have proposed different immunological mechanisms that explain the lower prevalence and severity of atherosclerosis in CD (24, 25). In CD, the chronic inflammatory immune response appears to be modulated in a way that limits the development of atherosclerosis, a phenomenon associated with an immunological profile marked by a predominance of M2-polarized macrophages, which are characterized by reduced pro-inflammatory activity and an enhanced capacity to resolve inflammation. M2-polarized macrophages are associated with lower production of pro-inflammatory mediators, such as tumor necrosis factor-alpha (TNF-*α*) and interleukin-6 (IL-6), which are essential for atherogenesis (26, 27). This may help explain why chronic inflammation in CD is less likely to promote atherosclerosis than would typically be expected in other chronic systemic inflammatory conditions. Furthermore, the trans-sialidase enzyme produced by *T. cruzi* has been shown to reduce inflammatory activity and the amount of atherosclerotic plaque in experimental models. However, this theory has never been demonstrated in humans.

Regarding EAT, our study showed that individuals with chronic CD have higher values than those reported in healthy populations from Brazil and other countries (28, 29), but these EAT values are similar to those observed in patients with clinical conditions such as hypertension, diabetes mellitus, and dyslipidemia, who are typically referred for echocardiography and carotid ultrasound (30). Similarly, Rodeles et al. have reported that the amount of EAT was increased in patients with chronic Chagas disease compared with controls without the disease, potentially due to metabolic alterations exacerbated by coexisting insulin resistance (31). Moreover, EAT is known to be strongly associated with pro-inflammatory activity and CAP (32). Further studies are needed to verify whether this association is related to the role of EAT as a possible immunological and nutritional niche favorable to *T. cruzi*, since host immunological regulation may lead to parasite reactivation and cause greater cardiac damage (31).

Our study also investigated the characteristics of EAT, CIMT, and CAP stratified by sex, age, and stage of CCC. Regarding sex, the results were consistent across all variables investigated (EAT, CIMT, and CAP). This can be explained by the inflammatory nature of CD, which may exert a greater influence than hormonal and metabolic differences between the sexes (33). On the other hand, as expected, individuals aged 65 years or older presented higher values of EAT and CIMT and a higher prevalence of CAP than younger individuals, pointing to a possible progressive accumulation of EAT and advancement of the atherosclerosis process with aging. The possible explanation for this difference may be related to the consequences of aging on the cardiovascular system, characterized by calcification of vascular walls and consequent greater accumulation of lipids, which contributes to the atherosclerosis process (34). For the comparison between the stages of CCC, the reduction in EAT in the advanced stages of CCC (with HF) can be explained by the more pronounced catabolic state observed in these individuals, leading to less systemic fat accumulation, as well as cardiac cachexia and cardiac remodeling, which decrease overall adipose tissue, including epicardial fat (35–37).

Regarding the association between PA levels and the studied variables, although statistically significant relationships were observed between higher PA levels and lower CIMT in the unadjusted analyses, such associations were not maintained in the model adjusted for potential confounding variables. Thus, the associations in the unadjusted model can be explained by the influence of confounding variables, especially age, which is usually associated with both PA levels and subclinical atherosclerosis (38, 39). The results of the association analyses in our study differ from some previous studies in the literature, because although inverse associations have been observed between PA levels and markers of subclinical atherosclerosis in populations at high cardiovascular risk (e.g., individuals with a history of cardiovascular disease or with the presence of risk factors such as arterial hypertension or dyslipidemia) (39, 40), our findings did not confirm such results in a population with CD. Furthermore, a previous study found that moderate-to-vigorous PA may reduce atherosclerosis in coronary and carotid arteries, particularly in elderly people with stable plaques (41). This contrasting finding lacks a clear consensus regarding its underlying causes. However, we speculate that the absence of an association in our study may be related to pathophysiological characteristics specific to CD, such as chronic inflammation and alterations in the autonomic nervous system, which may modulate the vascular effects of PA. These findings suggest that the management of cardiovascular risk in individuals with CD should adopt a broader approach—including the management of comorbidities in addition to PA recommendations.

This study has some limitations that should be considered. The cross-sectional design prevents the determination of causal relationships since exposure and outcome were assessed simultaneously. In addition, the sample included only patients regularly followed at the Evandro Chagas National Institute of Infectious Diseases (INI/FIOCRUZ) outpatient clinic, which may have biased our results toward the null hypothesis since these patients typically receive standard care for the treatment of comorbidities associated with worse cardiovascular health profiles. The assessment of PA levels using the IPAQ may have introduced measurement error, particularly among older adults due to recall and comprehension difficulties, potentially leading to non-differential misclassification and biasing the results toward the null hypothesis. In addition, obtaining images by echocardiography may be compromised by technical limitations, such as the presence of acoustic shadowing, suboptimal image quality, or patient anatomy, resulting in less accurate assessments of the cardiovascular health parameters under investigation. Although we adjusted for a comprehensive set of confounders based on a DAG, including age and stages of CCC, which may indirectly reflect disease duration, the lack of information on time since CD diagnosis may have resulted in residual confounding, as disease duration could influence both PA levels and atherosclerotic conditions. Future studies should include a longitudinal design, employ more accurate methods to assess PA, and utilize advanced imaging techniques to improve data quality.

## Conclusion

PA was not associated with markers of subclinical atherosclerosis in this population. These findings suggest that PA levels alone are not associated with markers of subclinical atherosclerosis in patients with chronic CD. The study highlights the need for multifactorial strategies that combine PA practice with the control of metabolic factors and comorbidities in this population.

## Data Availability

The raw data supporting the conclusions of this article will be made available by the authors, upon reasonable request.
