# Supplementary material for: Physical activity and subclinical atherosclerosis in chronic Chagas disease: a cross-sectional study
Source: Front Med (Lausanne). 2026 May 15;13:1793058. doi: 10.3389/fmed.2026.1793058 (PMC13219020; doi:10.3389/fmed.2026.1793058)

Supplementary Figure 1. The directed acyclic graph (DAG) illustrating the association between PA levels and EAT, CIMT, and CAP.


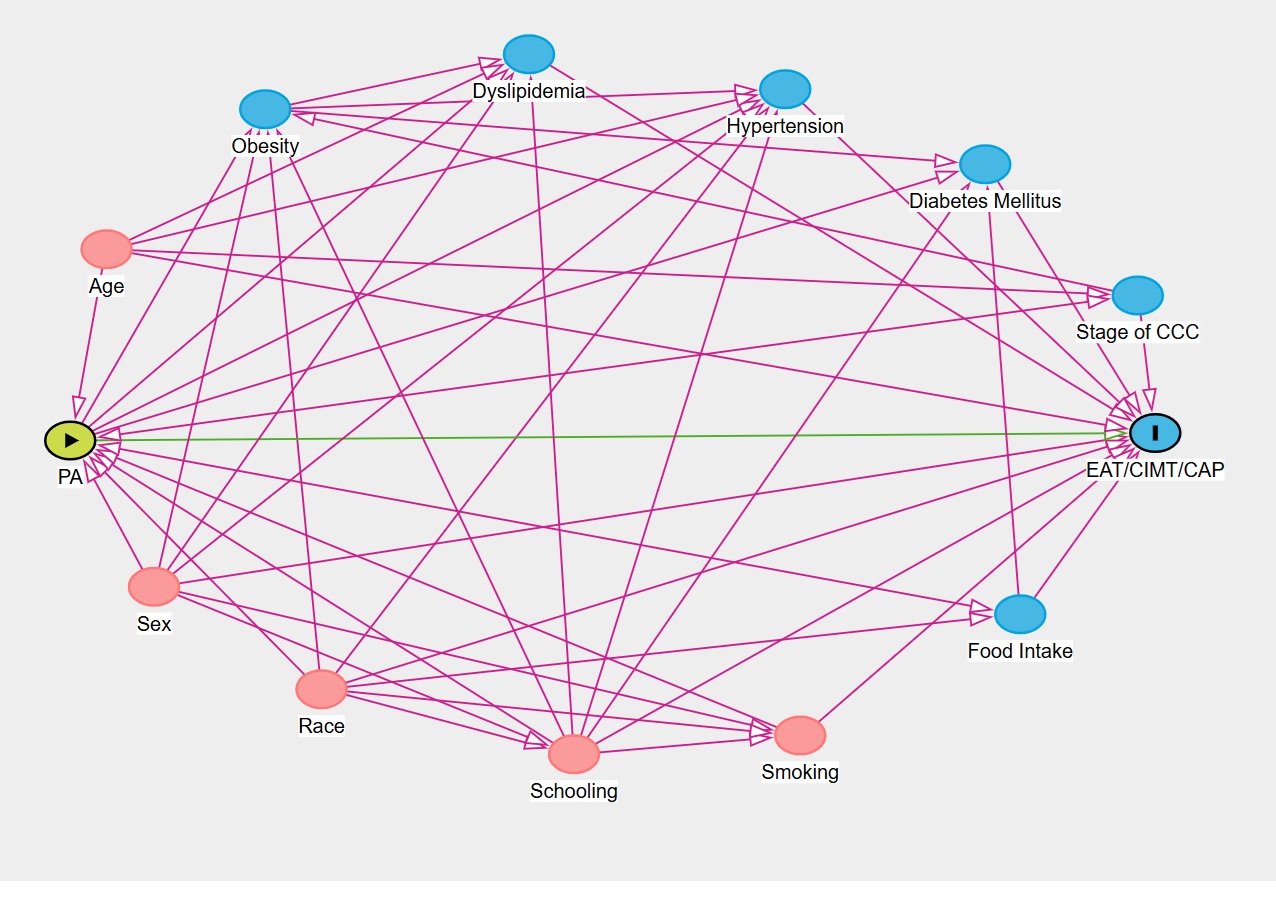


Supplementary Figure 2. Participant selection and eligibility flowchart.


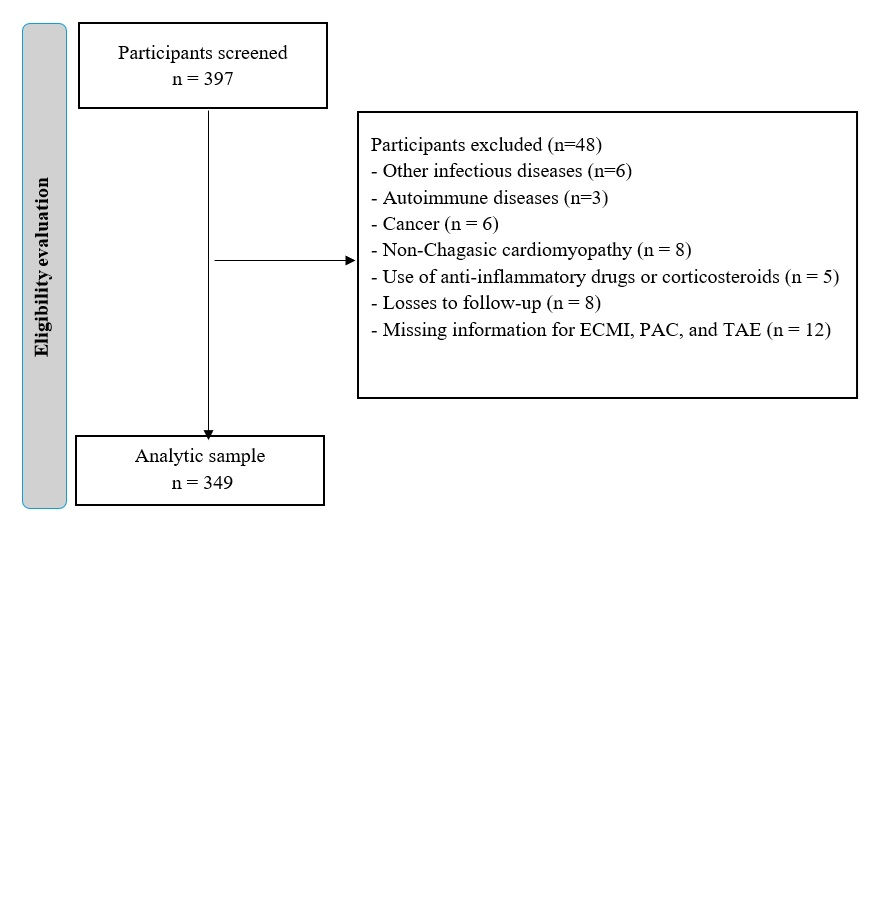

Supplement: Supplementary file 1 [file Table_1.DOCX]
